# Potential neuroprotective properties of epigallocatechin-3-gallate (EGCG)

**DOI:** 10.1186/s12937-016-0179-4

**Published:** 2016-06-07

**Authors:** Neha Atulkumar Singh, Abul Kalam Azad Mandal, Zaved Ahmed Khan

**Affiliations:** 1 Department of Integrative Biology, School of Biosciences and Technology, VIT University, Vellore, 632014 Tamil Nadu India; 2 Department of Biotechnology, School of Biosciences and Technology, VIT University, Vellore, 632014 Tamil Nadu India; 3 Centre for Interdisciplinary Biomedical Research, Adesh University, Bathinda, Punjab India

**Keywords:** EGCG, Neuroprotection, Neurodegenerative diseases, Antioxidant, Iron chelator, Cell signalling

## Abstract

Neurodegenerative diseases such as Alzheimer’s disease (AD) and Parkinson’s disease (PD) enforce an overwhelming social and economic burden on society. They are primarily characterized through the accumulation of modified proteins, which further trigger biological responses such as inflammation, oxidative stress, excitotoxicity and modulation of signalling pathways. In a hope for cure, these diseases have been studied extensively over the last decade to successfully develop symptom-oriented therapies. However, so far no definite cure has been found. Therefore, there is a need to identify a class of drug capable of reversing neural damage and preventing further neural death. This review therefore assesses the reliability of the neuroprotective benefits of epigallocatechin-gallate (EGCG) by shedding light on their biological, pharmacological, antioxidant and metal chelation properties, with emphasis on their ability to invoke a range of cellular mechanisms in the brain. It also discusses the possible use of nanotechnology to enhance the neuroprotective benefits of EGCG.

## Introduction

**Fig. 1:**
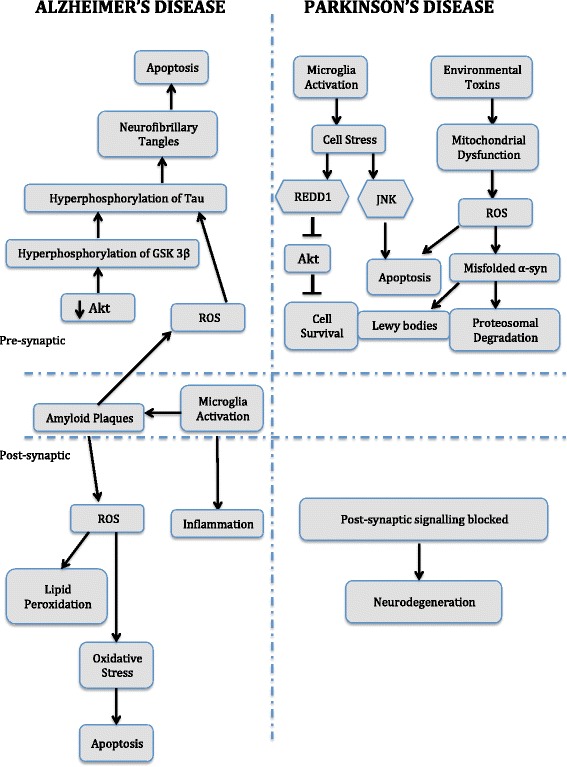
Proposed mechanism of Neurodegeneration in Alzheimer’s Disease and Parkinson’s Disease. Abbreviations: Akt – is another name for protein kinase B, GSK 3β – Glycogen synthase kinase 3 beta, JNK – c-Jun N-terminal kinases, Misfolded α-syn – Modified alpha synuclein, REDD1 – regulated in development and DNA damage responses 1, ROS – reactive oxygen species

Neurodegenerative diseases impose a significant social and economic burden. Since the population of developed countries are rapidly aging, age related disorders have become predominant. AD is the most common neurodegenerative disease with projected prevalence figures of 81 million people by 2040 [1]. It clinically characterized by the presence of extracellular amyloid plaques and intracellular neurofibrillary tangles that instigate the selective loss of neurons in the cerebral cortex and hippocampus through several mechanisms. Proposed mechanisms include microglia-triggered inflammation, over activation of glutamate receptors, increased intracellular calcium levels, generation of nitric oxide species, release of free radicals, mitochondrial dysfunction, synaptic dysfunction and loss [2]. PD on the other hand is the second most common neurodegenerative disease with projected prevalence figures of 7.1 million people by 2025 [1]. It is clinically characterized by the presence resting tremors, bradykinesia and rigidity triggered through dopaminergic neuronal loss in the substantia nigra. An important feature of PD is the presence of lewy bodies that are mainly composed of ubiquinated α-synuclein, neurofilament, synaptic vesicle protein and parkin. These lewy bodies trigger multiple mechanisms in the brain including mitochondrial dysfunction, release of free radicals, generation of nitric oxide species, JNK pathway activated apoptosis, microglia-triggered inflammation and disruption of protein degradation pathways [2] (Fig. 1).

Currently there is no effective treatment for either disease. As marketed therapeutic drugs are predominantly symptom-oriented with multiple side effects, where the adversity of the side effect increases in a dose dependent manner. They are therefore useful as long as their benefits outweigh any side effect [3]. Other highly specific interfering drugs currently being studied also do more harm than good, for instance, if we block signal peptidases for amyloid precursor processing to prevent plaques, we end blocking the other functions of the said secretase in the process [4]. Therefore there is a need to develop therapeutic agents with lower side effects and a broader spectrum of targets to not only treat the symptoms but also potentially reverse the pathology of the disease.

In the last decade, green tea polyphenols particularly its active component EGCG has gained a lot of attention as a potential therapeutic agent for preventing neurodegenerative [5, 6], inflammatory diseases [7] and cancer [8, 9] mainly due to their beneficial effects on human health. This ability is mostly attributed to their antioxidant [5, 6], radical scavenging [6], metal chelating [6, 9], anti-carcinogenic [9], anti-apoptotic [5, 6, 10] and anti-inflammatory properties [7]. Extensive research on EGCG have brought into light their potential to promote healthy ageing by improving the morphologic and functional alterations that occur in a natural ageing brain, their ability to suppress cognitive dysfunction [11], increase the learning ability [12] and reduce oxidative damage in the brain [12, 13].

Studies with PD have reported EGCG’s potential to attenuate apoptosis, supress accumulation of reactive oxygen species and free intracellular calcium, alter signalling pathways, lower nitric oxide levels and reduce oxidative stress [5]. While in case of AD, inhibition of reactive oxygen species accumulation, promotion of beta amyloid degradation, reduction in the production of beta amyloid, lower levels of beta and gamma secretase activity, higher levels of alpha secretase activity and suppression in phosphorylation of tau protein has been noted [14].

Therefore, in line with this evidence, added attention is being paid towards studying the neuroprotective and neurorescue roles of EGCG, in addition to their antioxidant, metal chelation and radical scavenging properties [14]. Important advances have been made in understanding the molecular events that cause the decline of signal transduction in neurodegenerative diseases and the role that EGCG plays in the modulation of these signalling pathways, particularly, their effect on cell death, survival genes [15] and signalling pathways such as mitogen activated protein kinase (MAPK), protein kinase C (PKC), protein kinase A and phosphatidylinostide 3-OH kinase/AKT pathways [16, 17]. So, here we will focus on the role of EGCG and its molecular mechanisms of neuroprotective action.

## Current status of knowledge

### Green tea polyphenols

**Fig. 2:**
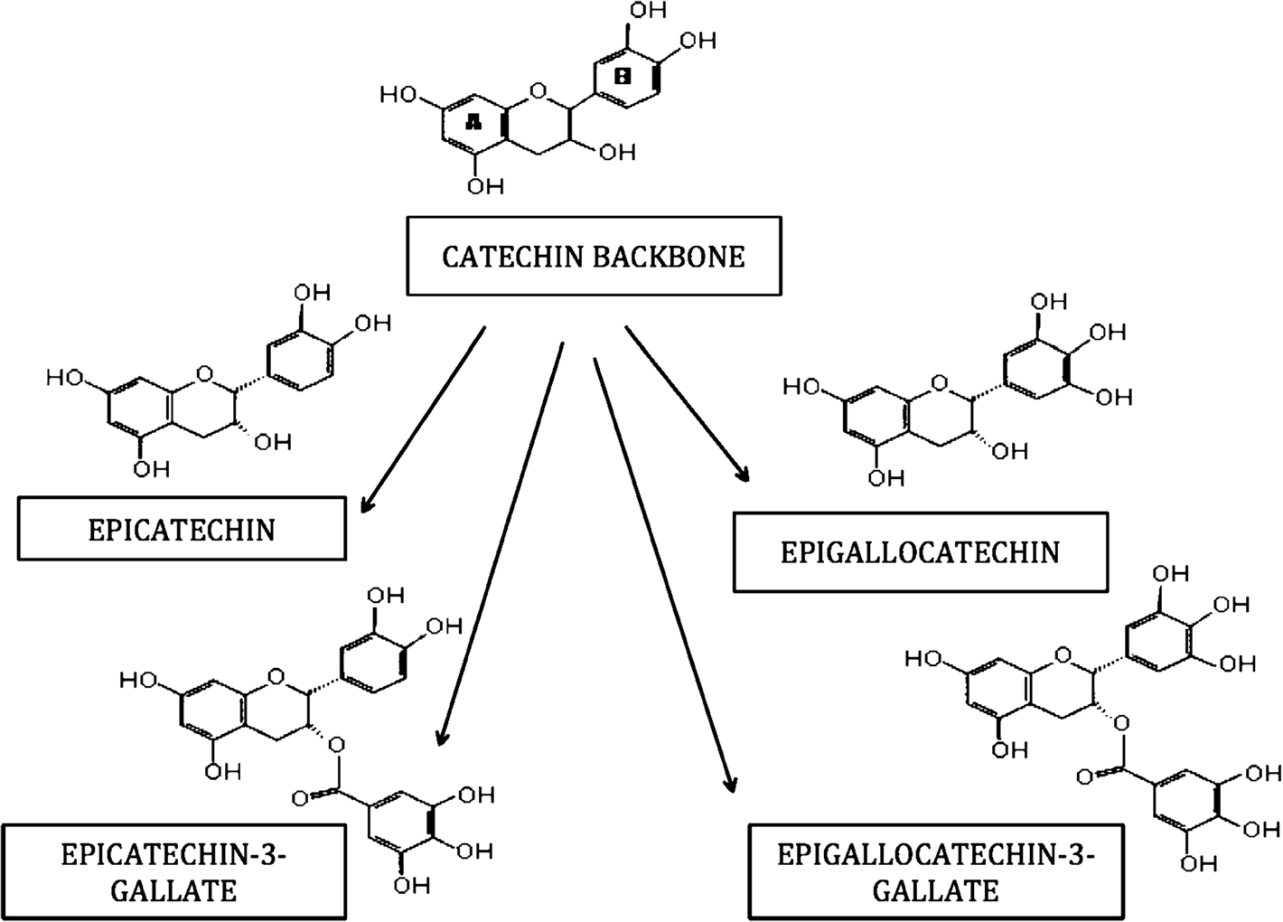
Structure of Green Tea Catechins and its four derivatives. Namely, Epicatechin, Epigallocatechin, Epicatechin-3-Gallate and Epigallocatechin-3-Gallate

Green tea is a traditional drink made from Camellia sinesis plant, widely consumed in Asian countries [18]. They are broadly made up of 4 derivatives based on their structural variations, including; epicatechin (EC), epigallocatechin (EGC), epicatechin gallate (ECG) and epigallocatechin-3-gallate (EGCG) (Fig. 2). Where, EGCG accounts for about 10 % of the extract dry weight [18, 19] and 50–80 % i.e. 200–300 mg in a brewed cup of green tea [20].

A few human studies have looked into the beneficial effect of tea consumption and have reported an inverse dose response relation between green tea consumption and cognitive dysfunction in dementia, AD and PD [21, 22]. Case controlled studies in Japan and United states have shown that consumption of 2 or more cups of tea per day reduced the prevalence of cognitive impairment [23, 24] and decreased the risk of PD [25, 26]. In support to this finding a 13 years long Finnish study with 30,000 adults aged 25–74 years also reported reduced risk of PD when 3 or more cups of tea were consumed per day [27]. In a more recent large scale cohort 20 year follow up analysis with approximately 50,000 men and 80,000 women, EGCG intake was found to be associated with 40 % lower PD risk [28]. Several case-controlled and cohort studies across North American, European and Asian populations have reported lower PD risk with tea consumption, however this effect was more significant in Asian populations [29]. Particularly, in Chinese populations a 28 % lower PD risk has been reported with tea consumption (3 cups/day for 10 years) [30]. These studies therefore suggest the presence of a relationship between tea consumption and lower rates of neurodegenerative disorders.

The metabolism of green tea polyphenols in the body has been also been studied. It was reported that green tea polyphenols are absorbed, distributed, metabolised and excreted from the body within 24 hours. In human studies, when 1.2 g of decaffeinated green tea was ingested, plasma levels ranged between 46 – 268 ng/ml within 1 hour of ingestion with cumulative excretion levels in the first 24 hours ranging from 1.6 – 3.2 mg [31]. With ingestion of five cups of tea in a day, the green tea polyphenol concentration in the plasma increased by twelve folds [8], which is enough to exert antioxidant activity against oxidative damage [32]. This data was further supported by animal studies, where administration 35 mg/kg/day of green tea polyphenols prevented not only oxidative damage and memory regression but also sufficiently delayed senescence [12].

EGCG has been reported to be more effective as radical scavengers when compared to vitamin E and C [19]. Within the derivatives the order of protective effects in vitro has been reported to be; ECG > EGCG > EC > EGC [33] and their order of antioxidant potential; EGCG ≥ ECG > EGC > EC [34]. The radical scavenging property has been attributed to the presence of an ortho-3’, 4’-dihydroxy moiety or an ortho-trihydroxyl group not their steric structures. In addition, with increase in the number of hydroxyls, the radical scavenging property becomes stronger, implying EGCG has stronger scavenging activity as they possess a trihydroxyl group in the B ring and also contain a galloyl moiety with three hydroxyl groups in the C ring [19].

In addition to the radical scavenging properties, EGCG also possess metal chelating properties. The two structures which give this compound its property of metal chelation include the ortho-3’, 4’-dihydroxy moiety and the 4-keto, 3-hydroxyl or 4-keto and 5-hydroxyl moiety [35]. These structures act as points of attachment for transition metals and neutralize their activity by converting their active form into a redox inactive complex to prevents oxidative damage of cells [36].

However, before EGCG’s role in neuroprotection can be established it is first important to determine whether EGCG is capable of crossing the blood brain barrier (BBB). In vitro studies with brain endothelial cell lines co-cultured with astrocytes have reported the successful diffusion of many flavonoids [37], which is also supported by in vivo studies. Oral administration of EGCG for a period of 5 days and EC for a period of 10 days has reported the presence of both compounds in brain tissue samples [38, 39]. Thereby implying that EGCG is capable of diffusing and localize in the brain.

### EGCG a potential therapeutic agent for neurodegenerative diseases

**Fig. 3:**
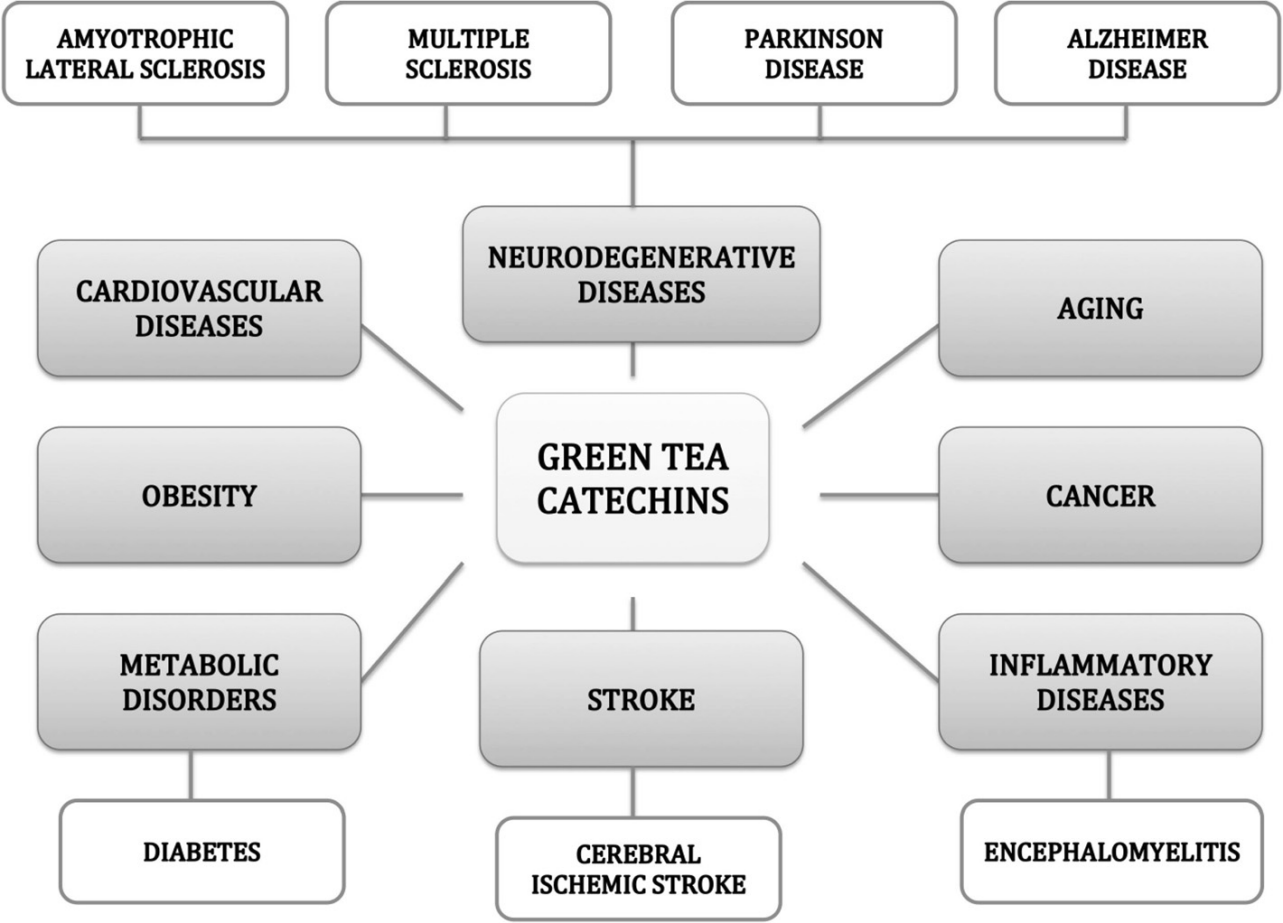
Green tea polyphenols – a potential therapeutic agent for Neurodegenerative Diseases, Aging, Cancer, Inflammatory Diseases, Stroke, Metabolic Disorders, Obesity and Cardiovascular Diseases

Neurodegenerative diseases are characterized by different structural and pathological conditions including the accumulation of modified or diseased proteins such as α-synuclein in PD [40], β-amyloid peptide and tau protein in AD [3, 41] that further contribute towards inflammation [42], elevate expression levels of pro-apoptotic proteins [43, 44], trigger glutamatergic excitotoxicity [45], iron accumulation [46] and oxidative stress [47]. It is therefore necessary to look for drugs capable of simultaneously manipulate multiple desired targets and exerting higher therapeutic effectiveness [48]. Since EGCG has a broad spectrum of biological and pharmacological activities, it can be measured as a much-anticipated therapeutic agent in the treatment of neurodegenerative diseases (Fig. 3) [49–54].

AD is an age-dependent neurodegenerative disease that instigates gradual deterioration of cognitive functions including memory loss and impairment in reasoning along with irreversible neuron loss. Neuropathologically, the hallmarks of AD include positive lesions such as amyloid plaques, neurofibrillary tangles, glial responses and cerebral amyloid angiopathy along with negative lesions such as neuronal loss and synaptic loss. The important biomarkers in AD pathology are considered to be phosphorylated tau protein (P-tau) and the 42 amino acid form of beta amyloid (Aβ_42_). Therefore agents that inhibit the formation of these 2 biomarkers can be used in the prevention of AD [55]. For instance in vitro studies, where neuronal cells were treated with 10 μM EGCG showed a protective effect against Aβ-induced cytotoxicity, either via the activation of the Akt signalling pathway [56] or by increasing the levels of acetylcholine, by EGCG acting as an acetylcholinesterase inhibitor [57]. While, the neuroprotective effects against Aβ-induced neuronal apoptosis were produced via its ability to efficiently scavenge reactive oxygen species [58]. EGCG has also shown the potential to inhibit Al (III) induced Aβ_42_ fibrillation and significantly reduce this Aβ_42_ fibrillation by preventing further conversion of Aβ_42_ monomers into a folded conformation. At the same time, it also has the ability to remold the preformed, mature, toxic fibrils into low toxic amorphous aggregates. Moreover, it could be implied that EGCG basically reverses the conformation of the complexes formed between Al (III) and Aβ42 in order to inhibit and remold Aβ_42_ fibrillation [Unpublished observations]. In addition to EGCG’s ability to reduce Aβ_42_ fibrillation, it also inhibits the aggregation of tau protein into toxic oligomers and at the same time remold existing oligomers to an unfolded monomeric state to rescue neuronal cells from tau-induced neurotoxicity [59]. EGCG administration in Alzheimer transgenic mice regulated the tau profile and markedly suppressed the phosphorylated tau isoforms [60]. This clearance of the phosphorylated tau isoforms occurred in a highly specific manner through the adaptor protein expression [61]. Furthermore, long-term oral administration of EGCG also reported significant improvement in spatial cognitive learning ability in rats [60, 62]. These studies therefore, demonstrate EGCG’s ability to reduce Aβ and tau toxicity and inhibit apoptosis, thus showing its potential for prevention of AD.

PD is the second most common neurodegenerative disease. It is characterized by features such as rigidity, postural instability and slowness of movement and tremors along with cognitive and psychiatric deficits. Neuropathologically, the hallmarks of PD include misfolding and aggregation of α-synuclein protein, damage and loss of dopamine (DA) neurons in the substantia nigra pars compacta (SN) along with oxidative stress caused due to mitochondrial dysfunction [63]. Therefore, agents that would target these hallmarks could be considered as important candidates for the treatment of PD. Oral administration of EGCG (2 and 10 mg/kg) in vivo, has shown to significantly reduce DA neuron loss in the SN and also prevent striatal DA and tyrosine hydroxylase protein level depletion [64]. In addition EGCG (200 μM) has also exhibited significant inhibitory effects against oxidative stress induced apoptosis [10]. These studies therefore suggest EGCG’s potential use as a therapeutic agent in the treatment of PD.

## Neuroprotective properties of EGCG

Green tea polyphenols are known to possess neuroprotective and neurorescue action. In particular, EGCG has shown to increase cell viability, decrease reactive oxygen species [65] and expression levels of endoplasmic reticulum stress markers and apoptotic markers [66, 67]. They also protect cells against mitochondrial dysfunction [68], 6-hydroxydopamine (6-OHDA) induced toxicity [69], apoptosis induced by mitochondrial oxidative stress [70] and glutamate excitotoxicity [71]. EGCG also preserves mitochondrial energetics [72] and limits the inflammation of the brain and neuronal damage [73, 74], which in turn prolongs the onset of symptoms, life span [75], cognitive skill and learning ability of the patient [62, 76, 77]. Not only does EGCG exert neuroprotective effects, it also wields neurorescue activity by promoting neurite growth [78]. Which, makes EGCG a good candidate for a potent disease-modifying agent with neurorescue and neuroprotective properties [79].

### Free radical scavenging and antioxidant action

Reactive oxygen and nitrogen species such as nitrite oxide, superoxide and hydroxyl free radicals are naturally produced to assist the host system in defence against oxidative stress and inflammation triggered through pathogens and infectious agents. But these species have a two faced nature, when overproduced in the body, they initiate a deleterious process making them a mediator to cell structure damage including DNA, proteins and lipids, which eventually leads to apoptosis and cell death [80].

Green tea polyphenols are biological antioxidants with radical scavenging properties. Among the green tea polyphenol family, EGCG and ECG are the most potent radical scavengers. This is attributed to the presence of an ortho-trihydroxyl group in the B ring [19], 4-keto and 5-hydroxyl group in the C ring i.e. the galloyl moiety [35] and the A ring in their structures. Also, the difference between antioxidant activities of EGCG and ECG is slight and is attributed to the number of hydroxyl groups each possesses [81]. In general they can scavenge 1,1-diphenyl-3-picrylhydrazyl radicals, peroxyl radicals [82], nitric oxide, lipid free radicals, singlet oxygen, peroxynitrite [83], hydroxyl free radicals and superoxide anion radicals [84] through three possible mechanisms. First, by chelating metal ions to their inactive forms [85]. Second, by direct interaction between catechins and peroxyl radical via a fast mechanism of electron (H-atom) transfer, to prevent DNA strand breakage [86]. And third, by preventing the deaminating ability of free radicals by forming stable semiquinone free radicals [87].

Oral administration of EGCG, in vivo has reported significant reduction in levels of lipid peroxidation products with elevated levels of enzymatic and non-enzymatic antioxidants [88]. Complete reversal of the damaging effects of AlCl_3_ on superoxide dismutase activity was noted along with markedly improvement in glutathione peroxidase, cytochrome C oxidase and acetylcholinesterase activity [89]. To better understand the antioxidant potential of EGCG, it was administered long-term in both young and old rats. Significant improvement in enzymatic and non-enzymatic antioxidant levels, 50 % reduction in the levels of malondialdehyde and 39 % reduction in protein carbonyl levels was also reported in both rats. Even with the reduction in dose from 100 mg/kg body weight to 2 mg/kg the same effects was observed [9, 90].

Consumption of green tea polyphenols in humans has shown to increase the antioxidant levels in the body. Long-term consumption of approximately 2-3 cups a day has reported an increase in both total antioxidant activity and total polyphenol content with a decrease in peroxide levels, glutathione levels and lipid hydroperoxide levels [91, 92]. Suggesting, that green tea polyphenols like EGCG could directly or indirectly regulate the antioxidant levels to reduce oxidative stress.

In addition to deterring oxidative stress, EGCG has also shown to hinder inflammation. It is a potent inhibitor of leukocyte elastase, which mediates the activation of matrix metalloproteinases (MMP) MMP-2 and MMP-9, which further trigger inflammation [93]. Oral administration of EGCG in vivo has also shown to significantly reduce inflammation in pulmonary fibrosis, block neutrophil mediated angiogenesis in inflammatory models [94] and also inhibit proinflammatory mediators such as myeloperoxidases in a dose dependent manner [95]. Implying that EGCG is a potent anti-inflammatory agent with therapeutic potential.

### Iron chelating activity

Iron accumulation is one of the major pathologies of neurodegenerative diseases causing neuron death at the site of iron accumulation [96]. This has generated a need for iron chelators such as EGCG. The mechanism of action involves the attachment of transition metal ions at two points: 3’4’-dihydroxy position in the B ring and the 4-keto, 3-hydroxy or 4 keto and 5-hydroxy in the C ring, which then inhibits the formation of transition metal catalyzed free radicals providing antioxidant and neuroprotective effects [35, 97]. Iron accumulation only occurs when the ionic iron participates in the Fenton reaction leading to the generation of reactive oxidative species, which triggers oxidative stress and activates the inflammatory cascade. In the process, activating primary and secondary messengers including cytokines (TNF α, IL-1 and IL-6), mast cells, histamine, transient receptor potential channels (TRP), acid sensing ion channels (ASIC), sodium channels and nuclear factor kappa-light-chain-enhancer of activated B cells (NF – kB) [98].

In the PD brain iron accumulation induces oxidative stress and reduction in the levels of neuromelanin that are visible well before the clinical manifestation of the disease develop, in vivo [99]. This accumulation of iron, directly or indirectly induces α-synuclein fibril formation, which then acts in concert with dopamine to induce the formation of lewy bodies and cause cell death [100]. However, in case of AD iron accumulation occurs in specific areas of the brain demonstrating it’s selective vulnerability of specific regions of the brain including the cerebral cortex, hippocampus and basal nucleus of meynert. Colocalization in lesions, plaques and neurofibrillary tangles along amyloid beta deposition; phosphorylation of tau protein and tangle formation was also noted. Since, these sites are centers of memory and thought, with the progression of the disease, in the form of neuronal death, these processes are gradually lost [101].

Treatment with EGCG for iron accumulation in AD and PD has portrayed its ability to regulate APP through the iron responsive element and at same time reduce the toxic levels of amyloid beta peptide [102]. Suppression of TNF – alpha and IL-1 beta levels along with inhibition of NF-kB activity was also noted in vitro, implying that EGCG’s iron chelating property plays an important role in these protective effects [103]. The kinetics and mechanisms of complex formation between iron and EGCG has reported that one molecule of EGCG is capable of reducing up to four iron (III) species [104]. In addition through interaction with Ngal, a biomarker for acute kidney injury, EGCG inhibits the chemical activity of iron by forming a stable Ngal-EGCG-Iron complex [105]. Making, EGCG a powerful metal chelating antioxidant.

### Lipid peroxidation

EGCG has been reported to protect from lipid peroxidation and DNA deamination by guarding cells from the initiators of lipid peroxidation i.e. t-butylhydroperoxide [106], 6-hydroxydopamine [107], iron [108], ultraviolet radiation [109], hydrogen peroxide [110] and 3-hydroxykynurenine [111]. In vivo studies designed to investigate the effect of EGCG on lipid peroxidation have reported a significant reduction in the extent of lipid peroxidation when thiobarbituric reactive substances (TBARS) levels were measured [112]. Along with a significant decrease in the levels of lipid peroxidation markers namely lipid hydroperoxides, 4-hydroxynoneal and malondialdehyde, with an increase in glutathione peroxidase activity and reduced glutathione concentrations [113]. Thereby, implying that EGCG is capable of protecting cells against lipid peroxidation.

### Modulation of cell signalling pathways, cell survival and death genes

EGCG protects not only through their antioxidant potential but also through the modulation of signalling pathways, cell survival and cell death genes. They interact directly with neurotransmitter receptor, downstream protein kinases or signalling cascades such as PKC, Akt and MAPK signalling pathways. This interaction between EGCG and the signalling cascade further dictates the response of the cell to environment or the stressor, ultimately leading to responses such as cell proliferation, apoptosis, synthesis of inflammatory mediators and neurite growth [114, 115].

#### Activation of PKC signalling pathway

PKC is the largest serine/threonine kinase family, making up almost 2 % of the human kinome. It is critical for normal cell growth [116] and plays an important role in the cell signalling machinery including its integral role in transduction pathways for hormones and growth factors. It also has an important role in the amalgamation of different types of memories [117]. An increase in PKC expression could potentially enhance memory, cognition and learning along with anti-dementia action [118] which, in turn would restore the normal PKC signalling. Consequently stimulation of neurotrophic activity, synaptic remodelling and synaptogenesis leads to the reduction of amyloid beta accumulation, tau hyperphosphorylation and apoptotic processes in the brain [119]. Making PKC activation in the neurons a prerequisite for neuroprotection [120].

The 12 isoforms of PKC are categorized into 3 subclasses based on their activators: conventional (α, β_I_, β_II_, γ), novel (δ, ε, θ, η, μ) and atypical (ι, λ, ζ). Here conventional isoforms are activated via phospholipids such as phosphatidylserine (PS), diacylglycerol (DAG) and Ca^2+^, whereas PS and DA activate novel isoforms as they lack Ca^2+^ binding site. Atypical isoforms are insensitive to both DAG and Ca^2+^ and are thus activated by phosphatidylinositols, phosphatidic acid, arachidonic acid and ceramide [121, 122]. The mechanism for PKC activation requires: first the phosphorylation of 3 distinct sites within the activation loop, turn motif and hydrophobic domain. Followed by binding of DAG and PS to promote the conformational activation of the proteins [123].

PKC isoforms are mainly targets for survival signalling. For instance, in vitro, EGCG effectively increases expression levels of PKC, in order activate the normal PKC signalling pathway. This activation offers neuroprotection against amyloid beta neurotoxicity, serum withdrawal, 6-OHDA and provides neurorescue action against neuronal cell damage [18, 78] along with rapid translocation and activation of Phospholipase D (PLD) in astroglioma cells [124]. Long term administration of EGCG has also shown to effectively protect against 6-OHDA and 1-methyl-4-phenyl-1,2,3,6-tetrahydropyridine (MPTP) toxicity, as enhanced expression levels of PKCα and PKCε have been observed. In addition, upregulation of previously depleted levels of PKCα has also been noted with further activation of Bcl – 2, signal related kinases – ERK 1, ERK 2 with a reduction in the levels of proapoptotic caspase 6, bax, bad, TRAIL and fas ligand [125]. Moreover, EGCG efficiently prevents the dissipation of the mitochondrial membrane potential with reduction in Bad levels [17, 126]. With PKC inhibition this protective effect was also abolished suggesting that these protective effects were PKC mediated.

In AD, amyloid beta fibrillation in particular was inhibited via the PKC signalling pathway. Where the fibrils generated due to the extracellular deposition of beta amyloid peptide, derived via the proteolytic cleavage of amyloid precursor protein (APP) by β and γ secretase instead of α secretase [127, 128] were inhibited when treated with EGCG in both cell and animal models. Transgenic studies have also suggested that these diminished levels of beta amyloid peptide and plaques stemmed due to the enhanced levels of PKC isoforms and α secretase expression [109, 129] which, implied that EGCG induced non-amyloidogenic sAPPα release and inhibited the generation of beta amyloid peptide via the PKC dependent activation of α secretase. To support this data transgenic studies with overexpression of PKCε reported significant decrease in beta amyloid peptide levels, plaque burden, reactive astrocytosis and neuritic dystrophy with increase in activity of endothelial converting enzyme that degraded the beta amyloid peptide and inhibited amyloid beta fibrillation [130].

In PD, treatment with EGCG has shown to induce a dose dependent inhibitory effect on DA presynaptic transporters (DAT) in the dopaminergic cells where PKC activation regulates DAT internalization and enhances synaptic DA levels. Also, this effect was completely abolished when PKC activation was blocked [131]. EGCG also inhibited the activity of catechol-O-methyltransferase (COMT) and in turn inhibited COMT catalysed methylation of endogenous and exogenous compounds, delivering a neuroprotective effect in both animal and cell models of PD [132].

#### Inhibition of MAPK signalling pathway

Mitogen activated protein kinases (MAPK) belong to a large family of serine/threonine kinases. They are important members of the signalling cascades involved in cell proliferation, inflammation, cytokine and inducible nitric oxide synthase expression [133]. MAPKs can be classified into 3 classes including ERK1/2 (p42/p44), c-Jun N-terminal kinase (JNK) and p38 [134]. Where ERK act as a determinant for cell growth, cell survival, motility, cell differentiation and pro-survival signalling [135]. JNK also known as stress-activated protein kinases (SAPK) maintains growth control and regulate apoptosis [136]. While p38 MAPK regulates cell cycle, cell death, inflammation, tumorigenesis, senescence and cell differentiation [137].

These classes of MAPKs have different modes of action through which they regulate their respective signalling cascades. For instance the activated ERK MAPKs regulate their signalling cascades through activation of cAMP response element binding protein (CREB) [138] and through upregulation of anti-apoptotic proteins [139]. While JNK MAPKs first become activated through an environmental stimulus followed by the activation of factors such as c-Jun, JunB, JunD and activating transcription factor 2 (ATF2) that help regulate the apoptotic-signalling cascade [136, 140]. In case of p38 MAPKs, cellular stressors, for example osmotic shock, inflammatory cytokines and growth factors are required for activation, which then phosphorylate transcription factors like ATF2, myc-associated factor X (Max) and myocyte enhancer factor 2 (MEF2) in order to regulate their respective signalling cascade in diseases like AD, rheumatoid arthritis and inflammatory bowel disease [137, 141].

Derivatives of green tea polyphenols have shown to interact with ERK, JNK and p38 pathways of MAPKs. For instance, in vitro treatment with EC effectively increased CREB and ERK phosphorylation along with significant increase in the mRNA levels of the glutamate receptor subunit (GluR2) and the GluR2 protein, which suggested that EC had the ability to regulate neurotransmission, plasticity and synaptogenesis [142]. Similarly, ECG through inhibition of p38 and ERK protected cells against H_2_O_2_ induced oxidative stress. At low concentrations EC reduced the activation of JNK [143]. Likewise EGCG induced cell death and increased cell survival in vitro [144] through the production of redox sensitive transcription factors like NF-kB, activator protein-1 (AP-1) and nuclear transcription factor erythroid 2p45 related factor (Nrf2), via the ERK pathway [145]. Furthermore, EGCG also triggered the expression of several antioxidant enzymes such as glutamate-cysteine ligase (GCL), haem oxygenase 1 (HO-1), manganese superoxide dismutase (MnSOD) and enhanced antioxidant response element binding (ARE) and the transcription activity of Nrf2, which in turn provided cells defence against oxidative stress [146] and neuron loss [147].

#### Activation of PI3K/Akt signalling pathway

One of the strongest pro-survival signalling systems is the PI3K/Akt pathway, which regulates many cellular responses and functions including cell survival, cell division, cell transformation [148], nutrient metabolism, myogenic differentiation and glycogen metabolism [149, 150]. In their activated form this pathway effectively blocks apoptosis. Conversely, when inhibited they accelerate apoptosis and abolish cell survival [151]. This mechanism involves first the activation of PI3K enzymes that catalyse production of phosphatidylinositol-3,4,5-triphosphate (PIP3). The activated form of PIP3 subsequently activates phosphoinositide-dependent protein kinase 1 (PDK1) [152], which in turn activates Akt and PKC isoenzymes [153]. The activated form of Akt maintains the inhibited state of glycogen synthase kinase 3β (GSK3β). With reduction in Akt activity, GSK3β hyper-phosphorylates [154], triggering tau accumulation in the brain, which further instigates the generation of neurofibrillary tangles (NFTs), ultimately causing neuron death [155].

Treatment with EGCG, in vitro has shown to activate PI3K/Akt pathway to inhibit cell death and increase cell survivability [156] via the suppression of apoptotic genes such as the Fas ligand, inhibition of GSK3β mediated tau protein hyperphosphorylation and through the enhanced expressions of all genes downstream of Nrf2 [157]. Moreover, it acts as a GSK3β inhibitor as it prevents hyper-phosphorylation of GSK3β via the activation of PIP3 and the PI3K/Akt pathway. EGCG also regulates the tau pathology through the suppression of phosphorylated tau isoforms [158]. Thus, affecting the downstream signalling cascade and preventing NFT generation [109, 159].

**Fig. 4:**
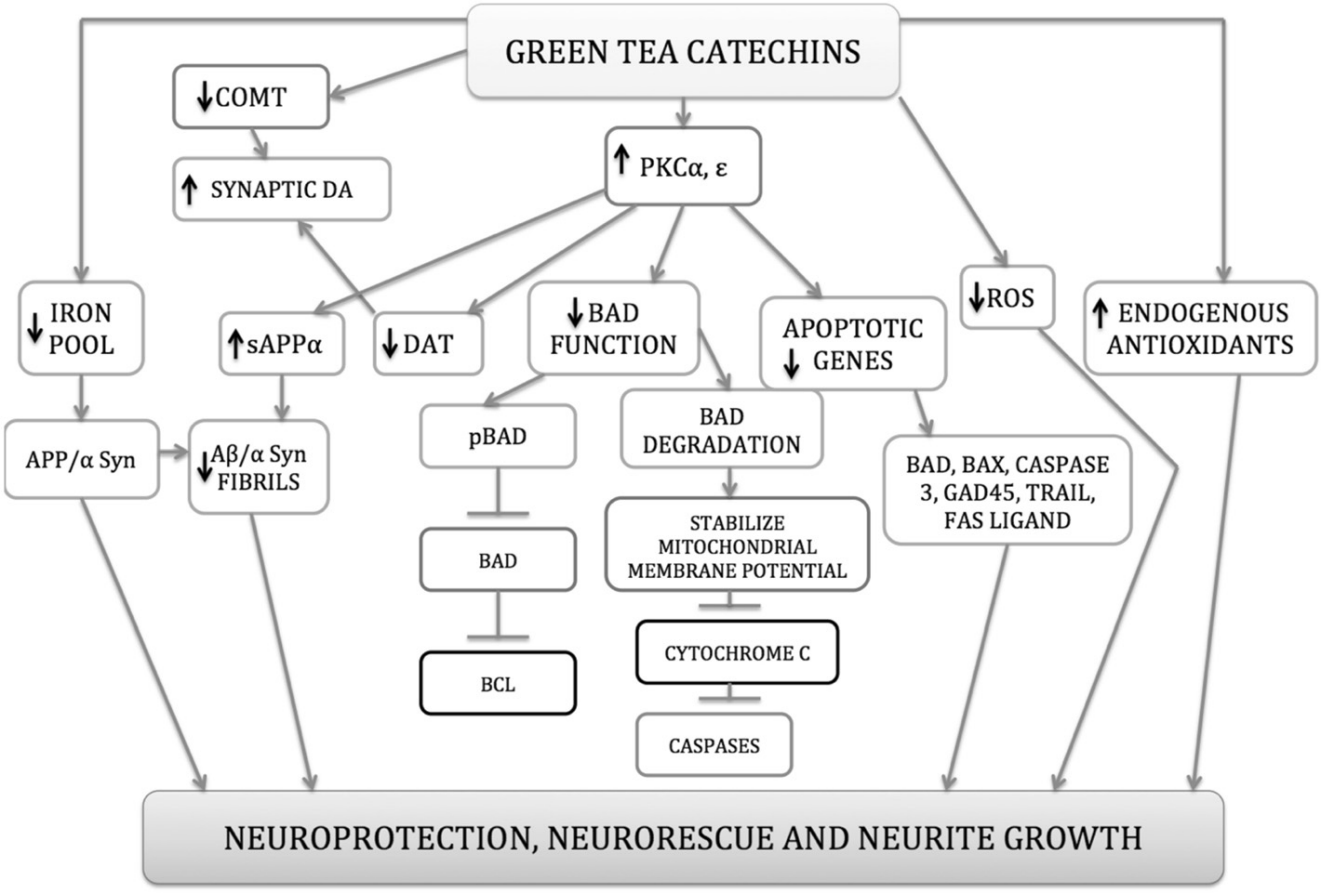
Proposed mechanism for Neuroprotection and Neurorescue action of EGCG. Abbreviations: α Syn – alpha synuclein, Aβ – amyloid beta peptide, COMT – catechol-o-methyl transferase, DAT – dopamine transporter, PKCα – protein kinase C alpha, PKCε – protein kinase C epsilon, ROS – reactive oxygen species and sAPPα – alpha secretase

Therefore, it can be hypothesised that EGCG affects neuronal survivability and cognitive performance via the activation of PKC and PI3K/Akt and the inhibition of the MAPK pathway. It also promotes neuronal communication, synaptic plasticity, angiogenesis and neurogenesis. On the other hand, via the inhibition of JNK and ASK-1 pathway, EGCG inhibits pro apoptotic-signalling and inflammation markers, which help in preventing neurodegeneration and aging. These specific interactions between EGCG and the signalling cascades consequently, increase strength of neuronal connections and expression of neuromodulatory proteins in the neurons. Thus increasing the expression of neuroprotection [145]. Hence, put together these studies have helped reveal novel pathways through which EGCG induces its neuroprotective effects (Fig. 4).

#### Modulation of cell death and cell survival genes

Modulation of cell survival and death genes via EGCG occurs in a dose dependent manner. For instance, treatment with low concentration of EGCG, both in vitro and in vivo models effectively lowers neurotoxicity via the reduction in the expression of pro-apoptotic genes; bax, bad, mdm1, caspase 1, caspase 6, TRAIL, p21, gadd45 and fas ligand with no effect on anti-apoptotic genes; bcl-w, bcl-2 and bcl-xL. Suggesting that EGCG induces this protective effect through the inactivation of cell death promoting genes rather than the up regulation of mitochondrial acting anti-apoptotic genes [54, 160–163]. Additionally, the decline in bad and bax expression activates secondary messengers including Ca^2+^, gangliosides, reactive oxygen species and stress kinases that further regulate the mitochondrial membrane permeabilization. This regulation in the membrane permeabilization increases the ratio of Bcl-2/Bcl-xL to Bax/Bad proteins, which heightens mitochondrial stability and protects the mitochondrial integrity [164, 165].

**Fig. 5:**
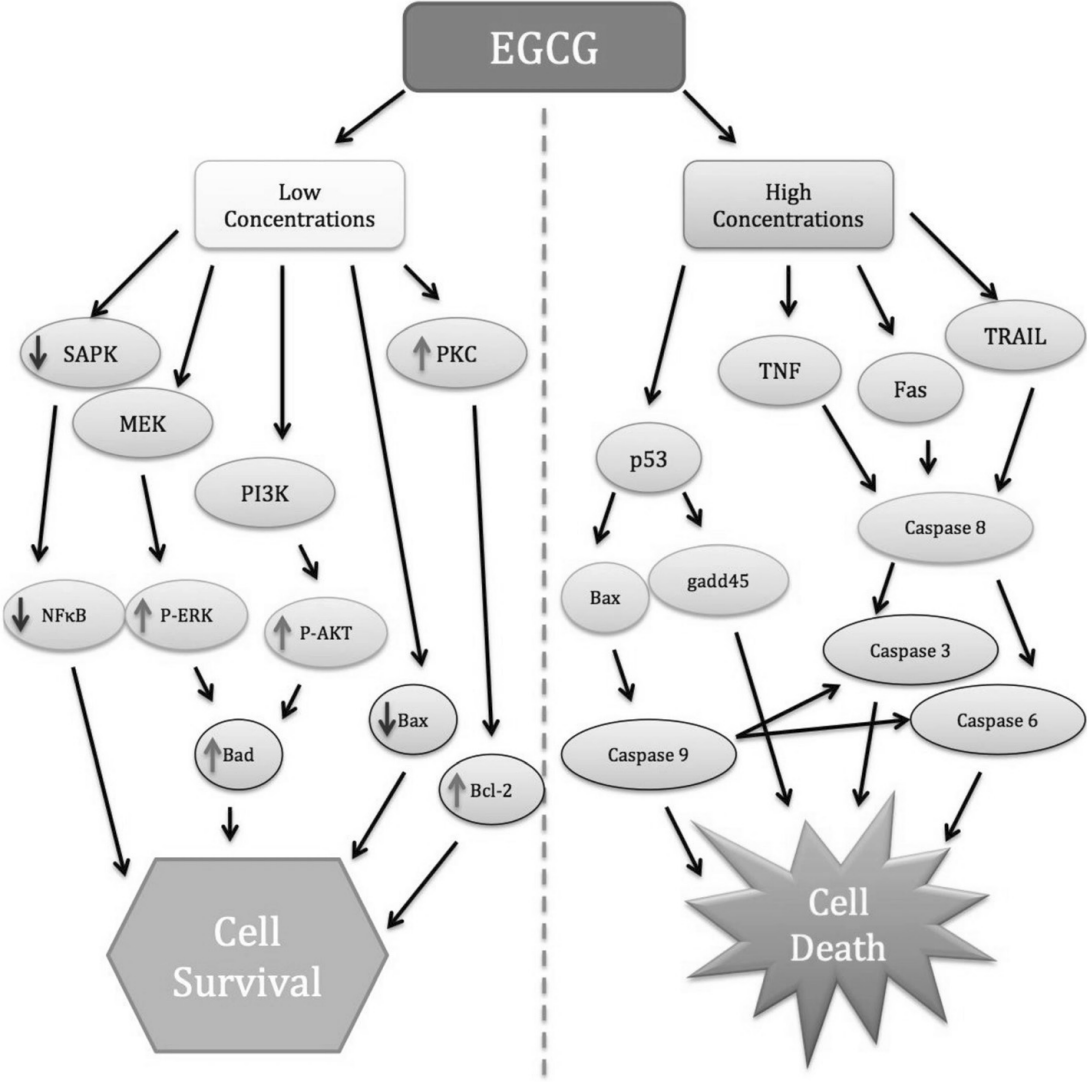
Overview of the possible gene targets involved in anti-apoptotic and pro-apoptotic actions of low and high concentrations of EGCG. Abbreviations: Akt – is another name for protein kinase B, ERK – extracellular signal-regulated kinase, MEK – is a member of MAPK signalling cascade, PI3K – phosphoinositide-3-kinase, PKC – protein kinase C, SAPK – stress activated protein kinase, TRAIL – TNF related apoptosis inducing ligand

Likewise, treatment with higher concentrations of EGCG induces a pro apoptotic or a pro toxic pattern of expression rather than an anti-apoptotic effect. In vitro higher levels of bax, bad, gadd45, p21/WAF1, fas, fas ligand, caspase 3, caspase 6 and caspase 10 expression were reported [161, 163]. Along with reduced expression levels of bcl-2, bcl-xL and bcl-w [15, 162]. This suggested that EGCG at higher concentrations in cell models, inhibited the cell survival genes and induced apoptosis, it also elevated p53 activity, activated caspase 9 and reduced expression of phosphorylated ERK 1/2, Bcl-2 and Bcl-xL proteins [166–168]. Thus proposing the first pharmacogenomics evidence of EGCG’s dose dependent mechanism of targeted action (Fig. 5). With support of this data highlighted through the years, we can consider EGCG as a good candidate for a therapeutic agent with neuroprotective properties (Table 1).

**Table 1:** Neuroprotective effects of EGCG in in vitro and in vivo models of neurotoxicity

Sr No	Mol. Mech	Drug	Model	Effect	Ref.
1	Antioxidant Effects	EGCG	• SOD1-G93A transgenic mice	• Regulates the expression of PI3-K, pAkt, and pGSK-3 signals • Reduces activation of NF-KB and caspase-3 • Prolongs the life span • Delays the onset of symptoms	[75, 175]
2	Antioxidant Effects	EGCG	• Age-associated oxidative damage in rat brain	• Increases activity levels of enzymic antioxidants like SOD and catalase • Increases activity of non-enzymic antioxidants like tocopherol and ascorbic acid	[90]
3	Antioxidant Effects	EGCG	• Glucose oxidase-induced neurotoxicity in H 19-7 cells	• Increases cellular resistance to glucose oxidase-mediated oxidative damage • Activates transcription factor Nrf2^a^	[176]
4	Antioxidant Effects	EGCG	• Glutamate-induced toxicity in HT22 mouse hippocampus neuronal cells • Kainic acid-induced neurotoxicity in rats	• Decreases glutamate-induced oxidative cytotoxicity • Inactivates NF-kB signaling pathway^b^ • Reduces ROS accumulation	[177]
5	Antioxidant Effects	Tea poly-phenol	• NMDA-induced neurotoxicity in mice	• Decreases ROS production	[178]
6	Antioxidant Effects	Tea poly-phenol	• Mice NMDA toxicity model	• Enhances behavioral and neurotoxic effects of NMDA • Decreases ROS production	[179]
7	Modulation of Signalling Pathways	EGCG	• 6-OHDA induced neurotoxicity in human neuroblastoma (NB) SH-SY5Y cell	• Modulates Akt^c^ signaling pathways	[69]
8	Modulation of Signalling Pathways	EGCG	• 6-OHDA induced neurotoxicity in human neuroblastoma (NB) SH-SY5Y cell	• Modulates Erk1/2^d^ pathway	[15]
9	Modulation of Signalling Pathways	EGCG	• Inflammatory response induced by IL -1β and Aβ [25–35] in human astrocytoma, U373MG cells	• Modulates NF-kB signaling pathway	[180]
10	Modulation of Signalling Pathways	EGCG	• Inflammatory response induced by IL -1β and Aβ [25–35] in human astrocytoma, U373MG cells	• Modulates MAPK^e^ signaling pathway	[180]
11	Modulation of Signalling Pathways	EGCG	• Long-term serum deprivation of human SH-SY5Y neuroblastoma cells	• Modulates HIF-1α^f^ pathway	[181]
12	Modulation of Signalling Pathways	EGCG	• β-amyloid (Aβ) induced toxicity in human SH-SY5Y neuroblastoma cells	• Modulates PKC^g^ pathway	[182]
13	Protective effect against protein aggregation	EGCG	• Long-term serum deprivation of human SH-SY5Y neuroblastoma cells	• Regulates APP • Reduced levels of toxic β-amyloid peptides in CHO cells over-expressing the APP “Swedish” mutation.	[102]
14	Protective effect against protein aggregation	EGCG	• MPTP- and DA-induced neurodegeneration in mice and rats	• Prevents the accumulation of iron and α-synuclein in the substantia nigra	[54]
15	Protective effect against protein aggregation	EGCG	• β-amyloid (Aβ) induced toxicity in human SH-SY5Y neuroblastoma cells. • C57/BL mice	• Increases PKCα and PKC levels • Enhances release of non-amyloidogenic sAPPα	[15]
16	Protective effect against protein aggregation	EGCG	• Biochemical assay	• Binds to β-sheet-rich aggregates • Converts mature α-synuclein and Aβ fibrils into smaller nontoxic aggregates	[183]
17	Protective effect against protein aggregation	EGCG	• Biochemical assay	• Inhibits amyloid-fibril formation	[184]
18	Protective effect against protein aggregation	EGCG	• Biochemical assay	• Inhibits the fibrillogenesis of both α-synuclein and Aβ • Promotes formation of nontoxic α-synuclein and Aβ oligomers. Prevents their conversion into toxic aggregates	[185]
19	Protective effect against protein aggregation	EGCG	• N2a cells stably transfected with “Swedish” mutant human APP	• Elevates active ADAM10 protein • Increases APP α cleavage and α-secretase activity • Produces no alteration in β-or γ-secretase activities	[186]
20	Protective effect against protein aggregation	EGCG	• PS2 transgenic mice model of AD	• Enhances memory function • Induces α -secretase activity • Reduces β- and γ-secretase activities	[187]
21	Modulation of cell death and survival genes	EGCG	• 6-OHDA induced neurotoxicity in neuroblastoma SH-SY5Y cells	• Decreases expression of pro-apoptotic genes like bax, bad, mdm1, caspase 1, caspase 6, TRAIL, p21, gadd45 and fas ligand. • No effect on the expression of anti-apoptotic genes like bcl-w, bcl-2 and bcl-xL	[162, 163]
22	Modulation of cell death and survival genes	EGCG	• 6-OHDA induced neurotoxicity in neuroblastoma SH-SY5Y cells	• Decreases expression levels of bcl-2, bcl-xL and bcl-w.	[162]
23	Modulation of cell death and survival genes	EGCG	• 6-OHDA induced neurotoxicity in neuroblastoma SH-SY5Y cells	• Increases expression levels of bax, bad, gadd45, fas, fas ligand, caspase 3, caspase 6 and caspase 10	[163]
24	Modulation of cell death and survival genes	EGCG	• MPTP and 6-OHDA induced toxicity in Male C57-BL mice	• Decreases bax, caspase-6, gadd45 and TRAIL expression levels	[54]
25	Modulation of cell death and survival genes	EGCG	• Head and neck squamous cell carcinoma (HNSCC) cells	• Decreases levels of Bcl-2 and Bcl-XL proteins • Increases Bax protein levels • Activates caspase 9	[166]
26	Modulation of cell death and survival genes	EGCG	• Human prostate carcinoma LNCaP cells	• Decreases expression of the proapoptotic protein Bcl-2 • Activates p21/WAF1, Bax and caspase 3.	[167]

^a^Transcription factor Nrf2 is a master regulator of the antioxidant response

^b^NFkB signalling pathway is the pathway which is activated in response to cell stress

^c^Akt signalling pathway promotes survival and growth in response to extracellular signals

^d^ERK1/2 cascade plays an important role in cellular proliferation, differentiation and survival

^e^MAPK signalling pathway plays an important role in cellular proliferation, differentiation and survival

^f^HIF – 1α plays an integral role in the body's response to low oxygen concentrations

^g^PKC signalling pathway regulates many cellular responses such as gene expression, cell proliferation, and inflammatory responses

## New treatment approach

Although EGCG is a good candidate for a neuroprotective agent due to its ability to manipulate multiple desired targets, its use as a therapeutic agent is limited. This is due to its poor availability, solubility and stability. Factors such as temperature, light, pH of the stomach, first pass metabolism, enzymes of the gut, interaction with food, insufficient absorption time and insufficient transport through the BBB limit the beneficial attributes of EGCG [169].

Nanotechnology based oral drug delivery systems could be employed to resolve these shortcomings easily. It is known that nanoparticles are generally non-toxic, size-controllable, produce fewer side effects and have high drug bioavailability and absorption capacity [170]. With particle sizes lower than 200 nm, these nanoparticles are capable of easily diffusing across the BBB [171].

To appraise the value of this new approach, our research group has delved into this subject. So far we have successfully encapsulated green tea polyphenols including catechin and EGCG into gold [172], casein [173], poly (D,L-lactic-co-glycolic acid) (PLGA) biopolymer [174] and polylactic acid (PLA) – polyethylene glycol (PEG) co-polymer nanoparticles [Unpublished observations], which are not only eco-friendly and biodegradable in nature but have also shown high drug bioavailability and absorption capacity. For EGCG PLA-PEG nanoparticles in particular, maximum drug entrapment of 96.25 % with the particle sizes ranging between 101.5 nm to 192.2 nm has been observed. Making their diffusion across the BBB possible. Furthermore, the release of the drug from the nanoparticles was also modulated to give a slow, sustained and controlled release for over 33 hours (under physiological conditions, pH 7.4). To protect against the pH of the stomach, first pass metabolism, enzymes of the gut and food interaction, a PEG coating on nanoparticles was introduced. 2 fold enhancement in neuroprotective properties such as antioxidant potential and metal chelation was observed, along with a significant improvement in the inhibition of Aβ_42_ fibrillation and remolding of toxic, insoluble Aβ_42_ oligomers was seen [Unpublished observations]. Therefore the encapsulation of EGCG into nanoparticles could not only help overcome all limitations of the pure drug but also enhance the neuroprotective effect of the agent. Making it a sensible option for oral drug delivery.

## Conclusion

The multi-etiological character of neurodegenerative diseases demands the need for the development of therapeutic agents capable of manipulating multiple desired targets. Green tea polyphenols, in particular EGCG is able to fulfil this criterion both in vitro and in vivo. EGCG has demonstrated good radical scavenging and metal chelation properties, in addition to its ability to invoke a range of cellular mechanisms including activation and inhibition of signalling pathways (PKC, MAPK and PI3K/Akt), enhancement of antioxidant action (radical scavenging, lipid peroxidation and production of endogenous defences), modulation of cell survival genes and cell death genes (anti-apoptotic action), neurite growth and bioenergetic action (stabilization of the mitochondrial potential), induction of iron-chelating effects on Aβ, tau and α-synuclein, elevation of synaptic DA (via COMT activity inhibition and DAT internalization), production of non-amyloidogenic sAPPα (by increasing α secretase levels for preferential APP processing) and inhibition of Aβ fibrillation, plaques, tau accumulation, NFT generation and α-synuclein fibrillation. These properties together give EGCG its neuroprotective and neurorescue abilities. Therefore, with the support from this data we propose EGCG as an iron chelating - brain permeable - antioxidant agent, which can modulate multiple brain targets. However, there is a need for examining this neuroprotective effect in depth through human clinical trials, since presently very few studies have delved into this subject.

## Abbreviations

6-OHDA: 6-hydroxydopamine
AD: Alzheimer disease
AP 1: Activated protein 1
APP: Amyloid precursor protein
ARE: Antioxidant response element binding
ASIC: Acid sensing ion channels
ATF2: Activating transcription factor 2
Aβ_42_: Beta amyloid
BBB: Blood brain barrier
COMT: Catechol-o-methyltransferase
CREB: cAMP response element binding protein
DA: Dopamine
DAG: Diacylglycerol
DAT: DA presynaptic transporter
EC: Epicatechin
ECG: Epicatechin gallate
EGC: Epigallocatechin
EGCG: Epigallocatechin gallate
GCL: Glutamate-cysteine ligase
GI: Gastrointestinal
GluR2: Glutamate receptor subunit
GSK3β: Glycogen synthase kinase 3β
HO-1: Haem oxygenase 1
JNK: c-Jun N-terminal kinase
MAPK: Mitogen activated protein kinase
Max: Myc-associated factor X
MEF2: Myocyte enhancer factor 2
MMP: Matrix metalloproteinases
MnSOD: Manganese superoxide dismutase
MPTP: 1-methyl-4-phenyl-1,2,3,6 tetrahydropyridine
NF-kB: Nuclear factor kappa-light chain enhancer of activated B cells
NFT: Neurofibrillary tangles
Nrf2: Nuclear transcription factor erythroid 2p45
PD: Parkinson disease
PDK1: Phosphoinositide-dependent protein kinase 1
PEG: Polyethylene glycol
PIP3: Phosphatidylinositol-3,4,5-triphosphate
PKC: Protein kinase C
PLA: Polylactic acid
PLD: Phospholipase D
PLGA: Poly (D,L-lactic-co-glycolic acid)
PS: Phosphatidylserine
P-Tau: Phosphorylated tau protein
SAPK: Stress-activated protein kinases
SN: Substantia nigra pars compacta
TBARS: Thiobarbituric reactive substances
TRP: Transient receptor potential channel
